# Cross-sectional relations of race and poverty status to cardiovascular risk factors in the Healthy Aging in Neighborhoods of Diversity across the Lifespan (HANDLS) study

**DOI:** 10.1186/s12889-016-2945-9

**Published:** 2016-03-14

**Authors:** Shari R. Waldstein, Danielle L. Beatty Moody, Jessica M. McNeely, Allyssa J. Allen, Mollie R. Sprung, Mauli T. Shah, Elias Al’Najjar, Michele K. Evans, Alan B. Zonderman

**Affiliations:** Department of Psychology, University of Maryland, Baltimore County, 1000 Hilltop Circle, Baltimore, MD 21250 USA; Laboratory of Epidemiology and Population Sciences, National Institute on Aging, Baltimore, MD USA; Department of Mathematics and Statistics, University of Maryland, Baltimore County, Baltimore, MD USA

**Keywords:** Race, Socioeconomic status, Poverty, Cardiovascular risk factors, Blood pressure, Lipids, Body composition, Glucose, C-reactive protein

## Abstract

**Background:**

Examine interactive relations of race and poverty status with cardiovascular disease (CVD) risk factors in a socioeconomically diverse sample of urban-dwelling African American (AA) and White adults.

**Methods:**

Participants were 2,270 AAs and Whites (57 % AA; 57 % female; ages 30–64 years) who completed the first wave of the Healthy Aging in Neighborhoods of Diversity across the Life Span (HANDLS) study. CVD risk factors assessed included body mass index (BMI), waist circumference (WC), total cholesterol (TC), high- and low-density lipoprotein cholesterol (HDL-C, LDL-C), triglycerides (TG), glycated hemoglobin (HbA1c), high-sensitivity C-reactive protein (CRP), and systolic, diastolic, and pulse pressure (SBP, DBP, PP). Interactive and independent relations of race, poverty status, and sex were examined for each outcome via ordinary least squares regression adjusted for age, education, literacy, substance use, depressive symptoms, perceived health care barriers, medical co-morbidities, and medications.

**Results:**

Significant interactions of race and poverty status (p’s < .05) indicated that AAs living in poverty had lower BMI and WC and higher HDL-C than non-poverty AAs, whereas Whites living in poverty had higher BMI and WC and lower HDL-C than non-poverty Whites. Main effects of race revealed that AAs had higher levels of HbA1c, SBP, and PP, and Whites had higher levels of TC, LDL-C and TG (p’s < .05).

**Conclusion:**

Poverty status moderated race differences for BMI, WC, and HDL-C, conveying increased risk among Whites living in poverty, but reduced risk in their AA counterparts. Race differences for six additional risk factors withstood extensive statistical adjustments including SES indicators.

## Background

Pronounced disparities associated with race and socioeconomic status (SES) are well documented with respect to cardiovascular disease (CVD) morbidity and mortality [1, 2]. In the United States, race-related disparities are most pronounced for African Americans (AAs) - associations that are thought to be partially [3] though not completely, accounted for by SES [2]. SES bears similarly potent relations to CVD morbidity and mortality that are not fully explained by race [4]. Race- and SES- related disparities in CVD morbidity and mortality may be partially attributed to differential burden of cardiovascular risk factors (e.g., hypertension, diabetes) including their frequency, severity, and/or control [5, 6].

It has long been emphasized that dual consideration must be given to race and SES within the context of health disparities research [2, 7]. Yet, disaggregating their respective influences has proven a challenging (and perhaps impossible) task. Williams and colleagues [7] suggest the need to explicitly examine the interaction of race and SES with respect to health outcomes to better understand their combined influence. Prior investigations have indeed noted significant interactive relations of race and SES to CVD risk profiles. In that regard, results of multiple investigations have shown associations of higher SES (e.g., education, income) with better CVD outcomes such as coronary heart disease, subclinical atherosclerosis, overweight and obesity, and various measures of inflammation in Whites but not Blacks [8–10].

It has further been noted that race- and SES-related disparities in CVD risk factors vary by sex [2]. For instance, higher levels of education have been associated with a poorer lipid profile in Black men, but a better profile in Black women [11]. In addition, Boykin and colleagues noted that lower SES was associated with greater hypertension, diabetes, body mass index, and smoking in Whites and in Black women, but findings were inconsistent for Black men [12].

Further investigation of race and SES interactions with respect to CVD risk is warranted for several reasons. First, a common challenge associated with work in this area is that the AA samples generally have a lower average SES than the White samples. Indeed, least common are samples that include lower SES, urban Whites. Furthermore, samples with a full spectrum of SES often focused solely on a single sex and/or racial group. Next, few studies of race by SES interactions have examined a broad CVD risk profile. Lastly, three way interactions of race, SES, and sex should be explicitly examined.

Accordingly, the present study aimed to extend prior work in this area by describing the interactive relations of race, poverty status, and/or sex with a series of anthropometric, metabolic, inflammatory, and hemodynamic CVD risk factors in a large sample of AA and White women and men of diverse SES following adjustment for a multitude of variables that may confound or partially mediate these associations- age, education, literacy, smoking, alcohol, and illicit drug use, depressive symptomatology, perceived healthcare barriers, select medication use, and select medical comorbidities.

## Methods

### Participants

Data were derived from the Healthy Aging in Neighborhoods of Diversity across the Lifespan (HANDLS) study - an epidemiological investigation uniquely designed to disentangle respective influences of race and SES, as indexed by poverty status, on a broad spectrum of age-related risk and disease outcomes including CVD risk factors [13]. The present participants were 1,301 AA and 969 White men and women who completed the first wave of the HANDLS study between 2004 and 2009. Participants were recruited from thirteen neighborhoods in Baltimore, MD, chosen to span diverse levels of income and socioeconomic status [13]. For HANDLS, approximately equal numbers of participants were recruited from separate clusters of contiguous census tracts – neighborhoods – containing sufficient numbers of residents to fill a factorial cross of sex, race, 5-year age groups, and poverty status (above or below 125 % of the Federal poverty guidelines based on household size). Neighborhoods were selected based on data from the 2000 census. Households were selected randomly for potential participation as were individuals within households. Participants were eligible if they self-identified as either Black/African American or White/Caucasian. Recruiters visited 32,959 dwellings in which they found 14,799 potentially eligible individuals in 9,904 households among whom 8,150 individuals actually met initial screening criteria. Of these potentially eligible individuals, 3720 participants met all study inclusion criteria and none of the following exclusion criteria: 1) outside of the age range 30–64 years; 2) currently pregnant; 3) within 6 months of receiving chemotherapy, radiation, or biological treatments for cancer; 4) diagnosed with AIDS; 5) unable to provide informed consent due to mental incapacity resulting from drug or alcohol intoxication, severe developmental disability, or dementia; 6) unable to provide at least five data measures on the mobile medical research vehicle (MRV); 7) without a verifiable address at time of consent, or lack of valid government issued identification; and 8) presence of uncontrolled high blood pressure (> 160/100). Seventy-eight percent of all eligible and non-excluded individuals agreed to participate in the first wave of the HANDLS study.

Of the 3,720 persons who completed a detailed initial interview in their home (study phase I), 2,707 returned to engage in a subsequent assessment on the MRV (study phase II).The present analyses targeted those 2,707 participants who had completed both phases of the HANDLS protocol (described in further detail below). We further excluded persons with a history of stroke (*n* = 60), dementia (*n* = 4), or other neurological disease (*n* = 105), heart failure (*n* = 60), HIV infection (*n* = 64), schizophrenia (*n* = 29), kidney disease or dialysis (*n* = 70), and those with missing data for key predictor variables (e.g., sociodeomgraphics; *n* = 142), An additional 150 individuals were excluded for providing a non-fasting sample for blood-based outcomes, and 215 persons were missing C-reactive protein (CRP). Imputation was performed for all outcome variables with < 10 % missing within each race, poverty status and sex subgroup (i.e., SBP, DBP, PP, BMI, WC, TC, LDL-C, HDL-C, TG). Multiple regression analysis (using age, sex, race, and poverty status as predictors) was used for imputation for the purpose of replicability. The final samples sizes for each outcome variable (after associated exclusions and increase in sample size following imputation) were: *n* = 2270 for SBP, DBP, PP, BMI, WC, TC, LDL-C, HDL-C, and TC; *n* = 2134 for HbA1c; and *n* = 2069 for CRP.

Phase I of the HANDLS protocol was administered in participants' homes and included screening, recruitment, informed consent, and administration of an interview concerning various sociodemographic factors, neighborhood characteristics, health care utilization and others. Phase II was conducted on MRVs parked in participants' neighborhoods. This visit consisted of a medical history, physical examination, laboratory measurements, cognitive testing, and other diagnostic procedures. All participants provided written informed consent. The study protocol was approved by the Institutional Review Board at the National Institute of Environmental Health Sciences. Participants were compensated $100 upon completion of phase II testing. HANDLS study data are available via formal request; see http://handls.nih.gov for associated form and application procedures.

### Measures

#### Demographic variables

Demographic characteristics included age (in years), sex (0 = female; 1 = male), self-identified race (0 = White; 1 = AA), and education (highest grade completed in school). Poverty status was predicated on size of household and reported family income relative to the 2004 Federal poverty threshold (e.g. $18,850 per year for a family of 4). Poverty status was defined as below 125 % of the poverty threshold (due to cost of living in Baltimore, MD), and non-poverty was defined as family income at or above 125 % of the poverty threshold. These data were dichotomized as 0 = not poverty and 1 = poverty. Poverty status was used as one of the primary selection criteria for HANDLS because pilot testing revealed that participants were better able to reliably report this status as opposed to a specific annual income.

#### Behavior/Lifestyle factors

Substance use was queried during the medical history. Alcohol status was coded as 0 = not current user (i.e., never tried, never used regularly, or used > 6 months ago) or 1 = used within the past 6 months. Smoking status was dichotomized as 0 = not current user (i.e., never tried, never used regularly, or former users) and 1 = current user. Use of marijuana, cocaine, and opiates was coded as 0 = not current user, or 1 = used in the past 6 months.

#### Psychological factors

Depressive symptoms were assessed via the Center for Epidemiological Studies-Depression scale [14], a 20-item self-report instrument that has been widely used and validated in community-based epidemiological studies. The reading subtest of the Wide Range Achievement Test – 3^rd^ Edition [15] was used as an estimate of literacy.

#### Healthcare barriers

Questionnaire items reflecting barriers to healthcare included access to specialty care, ability to get an appointment, expense, language differences, anxiety/fear, waiting too long, transportation, and excessive paperwork. Participants rated each as follows: 1 = major problem; 2 = minor problem, 3 = not a problem. Responses were reverse coded such that higher response value indicated greater problem; responses to all items were then summed.

#### Anthropometric measures

Height and weight were obtained using calibrated equipment, and BMI was computed as weight divided by height-squared (kg/m^2^). Waist circumference (WC) was measured to the nearest 0.1 cm with a flexible tape measure placed at the midpoint between the lower rib margin and the iliac crest at the end of exhalation during normal breathing.

#### Metabolic and inflammatory measures

Blood samples were obtained from an antecubital vein following an overnight fast. Levels of total serum cholesterol (TC), low and high density lipoprotein cholesterol (LDL-C; HDL-C), triglycerides (TG), and glycated hemoglobin (HbA1c) were assessed by standard laboratory methods at Quest Diagnostics (Chantilly, VA; http://www.questdiagnostics.com). TC, HDL-C, and TG were derived spectrophotometrically and LDL-C was calculated from HDL-C and TC. HbA1c was assessed via high performance liquid chromatography. All laboratory testing measures met or exceeded the standards set by Clinical Laboratory Improvement Amendments 1988, the U.S. Centers for Disease Control, and the Prevention-National Heart, Lung and Blood Institute Lipid Standardization Program guidelines with total allowable error (random and systemic) on a single result ranging from ≤ 8.9 % for TC to ≤ 15 % for TG. High sensitivity CRP levels were measured from blood samples by immunoassay at the National Institutes of Aging or Quest Diagnostics using similar equipment and reagents.

#### Hemodynamic measures

Systolic and diastolic blood pressure (SBP, DBP) were assessed using a standard brachial artery auscultation method following a 5 min rest in a seated position. Measures were obtained with the arm at a 90-degree angle, palm facing up. One measure was obtained in each arm; those measures were averaged. Pulse pressure was calculated by subtracting the mean of DBP from the mean of SBP.

#### Comorbidities

During the MRV visit, a HANDLS physician or nurse practitioner performed a comprehensive physical examination and medical history. The evaluator documented as unambiguously as possible any diagnosable medical conditions and use of medications. For the present analyses, select medical comorbidities were grouped into the following clusters: (a) coronary artery disease, myocardial infarction, peripheral artery disease, atrial fibrillation, angioplasty, carotid endarterectomy, and coronary artery bypass surgery as a cardiovascular disease (CVD) group; and (b) other prevalent metabolic/infectious/autoimmune (MIA) diseases including diabetes mellitus, thyroid disease, kidney disease, hepatitis, and systemic lupus erythematosus Medical conditions were dichotomized (0 = not present; 1 = present) and scores were summed across conditions within clusters.

### Data analyses

A series of ordinary least squares regression analyses were conducted to assess potential independent and interactive relations of race, poverty status, and sex to each cardiovascular risk factor. Regression models included the interactions of race x poverty status x sex, race x poverty status, race x sex, and poverty status x sex, in addition to the first-order terms of race, poverty status, and sex. Adjustment variables included age, use of alcohol, cigarettes, marijuana, cocaine, or heroine, an estimate of literacy, depressive symptomatology, perceived healthcare barriers, BMI (except for BMI and WC outcomes),and medical co-morbidity clusters (CVD and MIA; with diabetes removed from the MIA cluster for the HbA1c analyses). Medication use was included in the models as follows: use of antihypertensives, antilipidemics, and diabetes medications was included for the metabolic and inflammatory outcomes; antihypertensives were included for all blood pressure outcomes; and diabetes medications were included for the anthropometric outcomes. Distributions of the outcome variable and Q-Q plots of residuals were examined for normality.

## Results

### Sample characteristics

Descriptive statistics and frequencies were calculated for the final sample. See Table 1 for a summary of descriptive statistics in the full sample, and by race and poverty status.

**Table 1:** Descriptive statistics for full sample and by race/poverty subgroups

Variable	Full Sample (*n* = 2,270)	AA Poverty (*n* = 590)	AA Non-Poverty (*n* = 711)	White Poverty (*n* = 298)	White Non - Poverty (*n* = 671)
Age (years)	47.6 (9.3)	46.4 (9.1)	48.4 (9.3)	47.2 (9.5)	48.1 (9.4)
Female (%)	56.5	58.0	55.3	62.4	54.0
African-American (%)	57.3				
Below Poverty Line (%)	39.1				
Education (years)	12.5 (3.1)	11.7 (2.3)	12.9 (2.7)	11.4 (3.1)	13.4 (3.6)
WRAT-III Read score	42.3 (8.1)	39.4 (8.2)	41.4 (7.1)	41.8 (8.4)	45.9 (7.4)
CES-D score	14.6 (11.2)	16.4 (11.4)	12.6 (10.2)	17.8 (11.8)	13.6 (11.2)
Cigarette Use (%)	45.0	56.1	39.1	56.4	36.5
Alcohol Use (%)	54.9	52.2	55.0	50	59.2
Marijuana Use (%)	12.6	16.1	14.2	9.7	9.2
Cocaine Use (%)	5.3	8.6	4.4	7.1	2.7
Heroin Use (%)	3.2	5.4	2.7	4.2	1.2
CVD Cluster (%)	4.0	4.2	3.5	5.0	3.7
MIA Cluster (%)	23.1	22.5	22.2	27.5	22.5
Antihypertensives (%)	31.0	31.4	35.3	25.8	28.3
Antilipidemics (%)	16.0	11.7	15.1	16.8	20.6
Diabetes Medications (%)	9.3	8.5	10.8	10.1	8.2
Systolic BP (mmHg)	120.5 (17.4)	122.3 (17.9)	121.9 (16.3)	118.5 (19.1)	118.5 (17.0)
Diastolic BP (mmHg)	72.9 (10.6)	73.6 (11.2)	73.2 (10.4)	72.8 (11.2)	72.0 (10.2)
Pulse Pressure (mmHg)	47.6 (12.6)	48.6 (13.0)	48.7 (12.4)	45.7 (12.8)	46.4 (12.2)
Body Mass Index (kg/m^2^)	30.1 (7.7)	29.2 (8.1)	30.7 (7.4)	30.7 (8.6)	29.90 (7.3)
Waist Circumference (cm)	100.0 (17.5)	95.4 (17.7)	100.9 (16.1)	103.3 (19.0)	101.60 (17.3)
Total Cholesterol (mg/dl)	187.3 (41.9)	182.1 (40.2)	186.3 (42.3)	188.8 (43.5)	192.2 (41.6)
HDL-Cholesterol (mg/dl)	53.0 (17.0)	57.1 (19.8)	54.7 (16.8)	47.3 (13.6)	50.20 (14.6)
LDL-Cholesterol (mg/dl)	109.6 (35.3)	104.7 (35.9)	109.9 (35.0)	110.0 (34.7)	113.20 (34.75)
Triglycerides (mg/dl)	127.2 (125.3)	102.0 (55.8)	109.6 (83.7)	172.3 (244.5)	147.90 (119.7)
Glycated Hemoglobin (%)	6.0 (1.3)	6.1 (1.4)	6.1 (1.2)	5.9 (1.2)	5.80 (1.1)
C-Reactive Protein (mg/l)	4.8 (9.6)	5.7 (14.9)	4.6 (7.3)	4.8 (5.8)	4.30 (7.3)

Means and standard deviations unless otherwise stated. AA = African American; WRAT = Wide Range Achievement Test; CES-D = Center for Epidemiologic Studies Depression Scale; CVD = self-reported cardiovascular disease; MIA = self-reported metabolic/inflammatory/autoimmune disease; BP = blood pressure; HDL = high density lipoprotein; LDL = low density lipoprotein

### Regression analyses

There were no significant three-way interactions of race, poverty status, and sex or two-way interactions of poverty status and sex for any of the CVD risk factors (see Tables 2, 3 and 4). Results for significant interactions of race x poverty status and race x sex, and first order terms of race and poverty status are described below by conceptually related clusters of risk factors.

**Table 2:** Unstandardized regression coefficients and standard errors for all variables included in models examining anthropometric outcome measures

	Body Mass Index (mg/k^2^) (*n* = 2270)	*p*	Waist Circumference (cm) (*n* =2270)	*p*
Age	-0.02 (0.02)	0.273	0.05 (0.04)	0.186
Sex (0 = female; 1 = male)	-1.08 (0.56)	0.054	4.18 (1.27)	0.001
Race (0 = White; 1 = AA)	1.80 (0.53)	0.001	2.90 (1.21)	0.017
Poverty Status (0 = non-poverty; 1 = poverty)	1.35 (0.66)	0.041	3.00 (1.50)	0.046
Education	-0.24 (0.06)	0.000	-0.57 (0.13)	0.000
WRAT-III Read Score	0.02 (0.02)	0.330	0.03 (0.05)	0.618
Cigarette Use	-1.43 (0.21)	0.000	-2.47 (0.47)	0.000
Alcohol Use	-0.29 (0.20)	0.141	-0.27 (0.45)	0.553
Cocaine Use	-0.13 (0.34)	0.695	-0.26 (0.78)	0.743
Heroin Use	-1.47 (0.40)	0.000	-3.82 (0.92)	0.000
Marijuana Use	-0.34 (0.25)	0.182	-0.98 (0.58).	0.092
CES-D Score	0.01 (0.01)	0.582	0.05 (0.03)	0.157
Healthcare Barriers	0.03 (0.05)	0.546	-0.05 (0.11)	0.638
MIA Cormorbidity Cluster	0.96 (0.46)	0.036	2.59 (1.04)	0.013
CVD Comorbidity Cluster	-0.48 (0.79)	0.539	1.20 (1.79)	0.504
Diabetes Medications	5.36 (0.53)	0.000	13.03 (1.20)	0.000
Race x Poverty Status	-2.49 (0.84)	0.003	-7.82 (1.91)	0.000
Sex x Race	-2.06 (0.78)	0.008	-7.46 (1.77)	0.000
Sex x Poverty Status	-1.74 (1.03)	0.090	-3.95 (2.34)	0.091
Sexx Race x Poverty Status	1.15 (1.31)	0.377	2.70 (2.97)	0.364

AA = African American; WRAT-III = Wide Range Achievement Test third edition; CES-D = Center for Epidemiological Studies- Depression; MIA = metabolic/inflammatory/autoimmune disease; CVD = cardiovascular disease

**Table 3:** Unstandardized regression coefficients and standard errors for all variables included in models examining metabolic and inflammatory outcome measures

	Total Cholesterol (mg/dl) (*n* = 2134)	*p*	HDL - Cholesterol (mg/dl) (*n* = 2134)	*p*	LDL-Cholesterol (mg/dl) (*n* = 2134)	*p*	Triglycerides (mg/dl) (*n* = 2134)	*p*	Glycated Hemoglobin (%) (*n* = 2134)	*p*	C-Reactive Protein (mg/l) (*n* = 2069)	*p*
Age	0.62 (0.10)	0.000	0.21 (0 .04)	0.000	0.43 (0.09)	0.000	−0.37 (0.31)	0.233	0.01 (0.00)	0.000	0.02 (0.02)	0.517
Sex (0 = female; 1 = male)	−10.57 (3.32)	0.002	−12.27 (1.23)	0.000	−4.43 (2.78)	0.111	39.15 (9.78)	0.000	0.07 (0.08)	0.423	−2.06 (0.77)	0.007
Race (0 = White; 1 = AA)	−9.00 (3.15)	0.004	3.58 (1.16)	0.002	−6.04 (2.63)	0.022	−34.47 (9.26)	0.000	0.24 (0.08)	0.002	−0.41 (0.72)	0.569
Poverty Status (0 = non-poverty; 1 = poverty)	−5.08 (3.89)	0.192	−3.73 (1.44)	0.010	−3.62 (3.26)	0.266	15.06 (11.46)	0.189	−0.01 (0.10)	0.907	−0.77 (0.88)	0.383
Education	−0.80 (0.35)	0.022	0.24 (0.13)	0.068	−0.58 (0.29)	0.046	−1.87 (1.03)	0.069	−0.02 (0.01)	0.072	−0.09 (0.08)	0.282
WRAT-III Read Score	0.08 (0.13)	0.532	−0.04 (0.05)	0.421	0.12 (0.11)	0.279	−0.19 (0.39)	0.625	0.00 (0.00)	0.424	−0.01 (0.03)	0.824
Cigarette Use	−0.01 (1.25)	0.992	−2.42 (0.46)	0.000	1.08 (1.04)	0.301	12.07 (3.67)	0.001	−0.01 (0.03)	0.649	0.43 (0.29)	0.134
Alcohol Use	−0.93 (1.18)	0.432	2.41 (0.44)	0.000	−2.95 (0.99)	0.003	−5.04 (3.47)	0.147	−0.03 (0.03)	0.356	−0.05 (0.28)	0.853
Cocaine Use	1.47 (2.04)	0.472	2.79 (0.76)	0.000	−1.96 (1.71)	0.253	1.14 (6.02)	0.846	−0.07 (0.05)	0.159	−0.49 (0.46)	0.288
Heroin Use	−5.53 (2.45)	0.024	−0.54 (0.91)	0.554	−5.18 (2.05)	0.012	−3.25 (7.20)	0.651	0.03 (0.06)	0.645	0.67 (0.56)	0.230
Marijuana Use	−0.28 (1.51)	0.855	0.29 (0.56)	0.603	0.46 (1.27)	0.719	−5.01 (4.45)	0.261	−0.03 (0.04)	0.484	0.08 (0.34)	0.807
CES-D Score	−0.14 (0.09)	0.101	−0.02 (0.03)	0.481	−0.11 (0.07)	0.127	0.12 (0.25)	0.637	0.00 (0.00)	0.561	−0.02 (0.02)	0.269
Healthcare Barriers	−0.04 (0.28)	0.876	−0.12 (0.10)	0.245	0.08 (0.24)	0.727	0.66 (0.83)	0.426	−0.02 (0.01)	0.007	−0.14 (0.06)	0.028
Body Mass Index	−0.02 (0.13)	0.877	−0.67 (0.05)	0.000	0.37 (0.11)	0.000	1.73 (0.37)	0.000	0.02 (0.00)	0.000	0.30 (0.03)	0.000
MIA Cormorbidity Cluster	−8.38 (2.73)	0.002	−2.83 (1.01)	0.005	−5.76 (2.28)	0.012	−2.23 (8.03)	0.781	−0.06 (0.07)	0.356	0.28 (0.61)	0.653
CVD Comorbidity Cluster	−21.51 (4.82)	0.000	−4.42 (1.79)	0.013	−15.05 (4.03)	0.000	−13.43 (14.19)	0.344	−0.07 (0.12)	0.560	3.60 (1.06)	0.001
Antilipidemics	7.13 (2.74)	0.009	−2.10 (1.01)	0.039	1.27 (2.29)	0.580	53.78 (8.06)	0.000	0.22 (0.07)	0.001	−0.61 (0.62)	0.324
Diabetes Medications	−7.12 (3.36)	0.035	−1.48 (1.25)	0.236	−11.33 (2.81)	0.000	35.18 (9.90)	0.000	2.16 (0.08)	0.000	0.10 (0.75)	0.893
Race x Poverty Status	2.57 (4.96)	0.604	3.94 (1.84)	0.032	1.71 (4.15)	0.680	−18.58 (14.60)	0.203	0.07 (0.12)	0.595	1.12 (1.14)	0.325
Sex x Race	7.33 (4.64)	0.115	1.88 (1.72)	0.274	6.76 (3.89)	0.082	−9.14 (13.67)	0.504	0.01 (0.11)	0.914	0.78 (1.06)	0.462
Sex x Poverty Status	4.69 (6.12)	0.443	3.78 (2.27)	0.095	1.69 (5.12)	0.741	17.51 (18.01)	0.331	0.17 (0.15)	0.268	1.05 (1.38)	0.446
Sex x Race x Poverty Status	−5.93 (7.78)	0.446	1.38 (2.88)	0.633	−5.88 (6.51)	0.366	−30.08 (22.90)	0.189	−0.22 (0.19)	0.251	1.12 (1.77)	0.525

WRAT-III = Wide Range Achievement Test third edition; CES-D = Center for Epidemiological Studies- Depression; MIA = metabolic/inflammatory/autoimmune disease; CVD = cardiovascular disease

**Table 4:** Unstandardized regression coefficients and standard errors for all variables included in models examining hemodynamic outcome measures

	Systolic Blood Pressure (mm Hg) (*n* = 2270)	*p*	Diastolic Blood Pressure (mm Hg) (*n* = 2270)	*p*	Pulse Pressure (mm Hg) (*n* = 2270)	*p*
Age	0.48 (0.04)	0.000	0.02 (0.03)	0.382	0.45 (0.03)	0.000
Sex (0 = female; 1 = male)	5.44 (1.23)	0.000	4.79 (0.80)	0.000	0.66 (0.89)	0.461
Race (0 = White; 1 = AA)	3.57 (1.17)	0.002	0.88 (0.76)	0.250	2.69 (0.85)	0.002
Poverty Status (0 = non-poverty; 1 = poverty)	0.41 (1.45)	0.779	1.42 (0.94)	0.133	−1.01 (1.05)	0.336
Education	−0.25 (0.13)	0.050	−0.14 (0.08)	0.093	−0.11 (0.09)	0.230
WRAT-III Read Score	−0.01 (0.05)	0.881	−0.01 (0.03)	0.827	−0.00 (0.04)	0.991
Cigarette Use	−0.57 (0.46)	0.215	−0.81 (0.30)	0.007	0.24 (0.33)	0.464
Alcohol Use	0.29 (0.44)	0.511	0.35 (0.28)	0.216	−0.06 (0.32)	0.839
Cocaine Use	0.31 (0.75)	0.677	0.62 (0.49)	0.203	−0.31 (0.54)	0.570
Heroin Use	−2.44 (0.89)	0.006	−1.27 (0.58)	0.029	−1.17 (0.64)	0.069
Marijuana Use	2.34 (0.56)	0.000	0.77 (0.36)	0.034	1.57 (0.41)	0.000
CES-D Score	0.01 (0.03)	0.840	0.01 (0.02)	0.782	0.00 (0.02)	0.975
Healthcare Barriers	−0.03 (0.10)	0.749	−0.06 (0.07)	0.355	0.03 (0.08)	0.697
Body Mass Index	0.50 (0.05)	0.000	0.21 (0.03)	0.000	0.29 (0.03)	0.000
MIA Cormorbidity Cluster	0.44 (1.01)	0.659	−0.18 (0.65)	0.785	0.62 (0.73)	0.393
CVD Comorbidity Cluster	−2.88 (1.74)	0.100	−2.98 (1.13)	0.009	0.11 (1.26)	0.932
Antihypertensives	4.87 (0.80)	0.000	3.01 (0.52)	0.000	1.85 (0.58)	0.001
Race x Poverty Status	2.27 (1.84)	0.218	0.75 (1.20)	0.530	1.52 (1.33)	0.255
Sex x Race	−2.62 (1.71)	0.126	−0.36 (1.12)	0.745	−2.26 (1.24).	0.069
Sex x Poverty Status	0.15 (2.25)	0.947	−1.09 (1.47)	0.457	1.24 (1.63)	0.447
Sex x Race x Poverty Status	−1.10 (2.86)	0.700	−1.55 (1.86)	0.404	0.45 (2.07)	0.828

WRAT-III = Wide Range Achievement Test third edition; CES-D = Center for Epidemiological Studies Depression; MIA = metabolic/inflammatory/autoimmune disease; CVD = cardiovascular disease

### Anthropometric measures (BMI, WC)

**Fig. 1:**
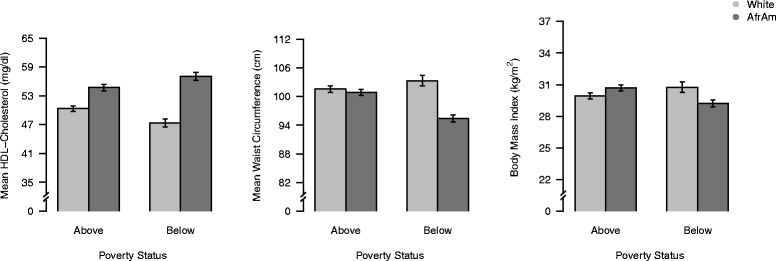
Interactive relations of race and poverty status to body mass index, waist circumference, and HDL-cholesterol; predicted values and standard errors after adjustment for age, education, literacy, substance use, depressive symptoms, perceived health care barriers, medical co-morbidities, and medications

Results revealed **s**ignificant interactions between race and poverty status for BMI and WC (see Table 2 and Fig. 1). Specifically, non-poverty AAs had greater BMI and WC than those living in poverty. However, non-poverty Whites had lower BMI than those living in poverty. Additionally, for those living in poverty, BMI was higher for Whites than for AAs, whereas BMI was higher for non-poverty AAs than for non-poverty Whites. Furthermore, AAs living in poverty had lower WCs than non-poverty AAs, as well as Whites regardless of poverty status.

**Fig. 2:**
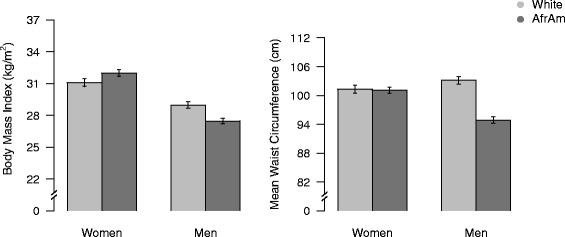
Interactive relations of race and sex to body mass index and waist circumference: predicted values and standard errors after adjustment for age, education, literacy, substance use, depressive symptoms, perceived health care barriers, medical co-morbidities, and medications

Significant race by sex interactions were also found for BMI and WC. Specifically, AA women had greater BMI than White women, whereas AA men had lower BMI than White men (see Fig. 2). In addition, White men had a higher WC than AA men, but there was no difference in WC between AA and White women

### Metabolic and inflammatory measures (TC, LDL-C, HDL-C, TG, HbA1c, CRP)

Regression analyses for lipid measures showed significant interactions between race and poverty status for HDL-C, but not TC, LDL-C, or TG (see Table 3 and Fig. 1). The interaction between race and poverty status was such that Whites living in poverty had lower HDL-C levels than non-poverty Whites, whereas AAs living in poverty had higher HDL-C than non-poverty AAs (see Fig. 1). Additionally, the first-order term of race was associated with TC, LDL-C, and TG, with AAs having lower lipids levels than Whites for each measure. Interactions of race and sex, and the first-order term of poverty status were not associated significantly with any of the lipid measures.

As shown in Table 3, there were no interactive effects of race and poverty status or race and sex for HbA1c. There was a significant main effect of race such that AAs had higher HbA1c than Whites. Poverty status was not associated with HgbA1c. Additionally, there were no interactive effects of race and poverty status or race and sex for CRP. Neither race nor poverty status was significantly associated with CRP.

### Hemodynamic measures (SBP, DBP, PP)

As shown in Table 4, there were no significant interactions of race and poverty status, or race and sex for SBP, DBP or PP. There was a significant main effect of race on SBP and PP such that AAs had higher resting SBP and PP than Whites. There were no significant main effects for poverty status for any blood pressure measure.

## Discussion

Estimating the respective influences of race and SES on CVD risk has proven a challenging task. Here we used data from the HANDLS study to examine potential interactive relations of race, poverty status, and sex to a broad spectrum of CVD risk factors within a socioeconomically diverse sample of urban-dwelling AA and White adults. Results revealed synergistic associations of race and poverty status for BMI, WC, and HDL-C. Whereas Whites living in poverty had greater BMI and WC than non-poverty Whites, AAs living in poverty had *lower* BMI and WC and greater HDL-C as compared to non-poverty AAs.

Although prior research with these outcome measures has more typically used education as the primary SES indicator, associated findings are similar to our own. For example, in contrast to White women, higher levels of education did not protect against the development of overweight and obesity among AA women [10]. This “lack of protection” from higher SES with respect to BMI has also been shown in AA adolescents [16]. Similarly, inverse relations between education and HDL-C have been noted in AAs [17].

These findings may be considered consistent with the "diminishing returns" hypothesis [18] which posits that AAs may not benefit as much as Whites from higher levels of socioeconomic resources. However, an alternative explanation is the known lack of equivalence of socioeconomic resources in AAs and Whites. Specifically, multiple SES-related variable differ systematically between AAs and Whites. For example, on average, AAs obtain less quality education than Whites [19]. Furthermore, AAs have smaller incomes (and less wealth) than Whites at each level of education [2]. Thus, SES measures likely have differential validity within racial/ethnic groups. Better understanding of respective influences of SES indicators on CVD risk factors within AA and Whites is needed.

Considered from the perspective of risk and resilience, another possibility is that lower SES confers greater risk in Whites than AAs with respect to BMI, WC, and HDL-C, whereas the opposite is true for higher SES. Complex explanatory mechanisms may differ within the race and SES subgroups. For example, AAs below poverty are more likely to suffer from food insecurity than their White peers also below poverty, whereas there is no difference between Whites and AAs above poverty [20]. Thus, one must consider that the lower BMIs and WCs among this group may, in part, reflect a larger proportion of underweight persons. Further, in the present sample, food insecurity was associated with worse diet quality in Whites than AAs [21]. Within White women, Montez and Zajakova [22] have reported increasing levels of all-cause and CVD mortality associated with lower levels of education in conjunction with declining levels among the more highly educated. Better understanding of the sources of these secular trends may point toward additional explanatory mechanisms.

Regarding those of higher SES, prior research has shown that AAs tend to report greater stress and discrimination than AAs of lower SES [23, 24]. Higher cortisol levels that accompany stress have been associated with increased appetite [25], and may be a relevant influence to consider. It is also well known that multiple dimensions of neighborhoods, such as access to healthy food and walkability, differ systematically for AAs and Whites even in the context of similar economic means [2]. However, associated risks tend to be greater for AAs than Whites. Examination of contextual influences specific to Baltimore City may be informative.

Distinct patterns of race associations (i.e., main effects of race) were found for six of the 11 risk factors - SBP, PP, TC, LDL-C, TG, and HbA1c. AAs had higher levels of SBP, PP, and HbA1c than Whites, whereas Whites had higher levels of TC, LDL-C, and TG than AAs. These findings are consistent with prior epidemiological studies which show that AAs have higher blood pressure, and greater fasting glucose, HbA1c, and risk for diabetes, but better cholesterol profiles than Whites [1, 26, 27]. The present findings reinforce that the above racial differences are not fully attributable to poverty status, education, or literacy, substance use, depressive symptoms, perceived healthcare barriers, comorbidities, and medication use (as measured herein). However, more detailed measurement of these adjustment variables (particularly substance use) is warranted as residual confounding seems likely. Nonetheless, continued evaluation of additional possible mediational pathways is needed. The present findings, and those in the literature, may suggest a role for a dysfunctional gene-environment interaction as well as the presence of other factors relevant to the basic biology of disadvantage and health disparities [28].

### Intersection of race and SES

Race is a complex biopsychosocial and sociopolitical construct [2, 7, 29] that reflects a multitude of influences ranging from geographic origin, ancestry, and culture (e.g., ethnicity), in addition to economic, political, and historical factors, environmental context, and racism. That the HANDLS participants self-identified as AA and White highlights the present view of race as a social (not a biological) construct. In the present study, associations of race with CVD risk remained after adjustment for multiple potential confounders and/or mediators. Vanderweele [29] offers an elegant discussion of interpretation of covariate adjusted race coefficients, highlighting the range of variables to which one might attribute remaining race associations, e.g., genotype, phenotype, individual/family SES, neighborhood SES. We suggest that the present findings further reflect, at least in part, the pronounced environmental and interpersonal stressors that are disproportionately experienced by AAs. Indeed, racial disparities in health have been shown to exist at every level of SES [2].

SES is another complex construct that has been indexed in various ways, the most common of which include measures of educational attainment, income, occupation, occupational prestige, home and goods ownership, and area-based resources [30, 31]. Each measure confers its own strengths and weaknesses, with none fully capturing the complexities of social disadvantage [30]. Further understanding of potentially unique contributions of each measure to health status may be important in the translation of research to intervention and policy. In addition, each type of measure may operate differently with respect to moderating (or partially mediating) relations of race/ethnicity to health outcomes [32]. It is striking that, in the present sample, poverty status (our primary SES indicator) moderated relations of race to only three of 11 CVD risk factors, presented greater risk for Whites than AAs, and showed no additional main effects with respect to these outcomes. However, findings may have been quite different had we used education as our SES indicator. Furthermore, prior work from HANDLS has shown differential relations of subjective SES to CVD risk profile in AAs and Whites [33]. Future work should contrast the respective (and cumulative) influences of distinct SES indicators in addition to specifically testing potential mediational pathways.

Both race and SES likely index many similar exposures to challenging environmental and psychosocial contexts [2, 34–36] that negatively impact health status. Yet, it remains unclear what health compromising (or health promoting) dimensions of self-identified race are distinct from SES and, conversely, what influences of SES are separate from race. We agree with Williams et al. [2] that the independent influences of race and SES on disease probably cannot be fully disentangled. However, future research could use various statistical methods to construct complex pathways from race and SES to disease outcomes [29].

Clear correlates of race include racism and discrimination [2]; yet, even those influences may vary by SES and sex. Various other mechanistic pathways to CVD outcomes may differ for AA and White men and women of higher versus lower SES. Increased consideration of such intersectionality has been strongly advised. Williams et al. [2] further note that multiple dimensions of social inequality, other than SES indicators, differ between AAs and Whites. These complex pathways range from micro- to macro- level factors. Macro level influences have been discussed thoroughly with examples including institutional racism, historical oppression, and access to quality health care. Relevant neighborhood characteristics include racial segregation, access to healthy food and green space, exposure to environmental toxins, pollution, noise, crowding, neighborhood physical and social disorder, and crime.

Both race and SES may further impact multiple psychosocial, lifestyle, psychophysiological, and biological factors (e.g., [2, 34–36]) such as social integration, social support, perceived stress, vigilance, anger, anxiety, symptoms of depression and post-traumatic stress disorder, substance use topography, physical activity, dietary patterns and preferences, sleep, stress physiology (e.g., autonomic, neuroendocrine, and immune function), and epigenetics.

### Study strengths and limitations

The present investigation has several notable strengths. First, the HANDLS study was explicitly designed to disentangle respective influences of self-identified AA and White race from poverty status, and provided a large and diverse sample of men and women that supported explicit examination of race, poverty status, and sex interactions. Second, our inclusion of multiple relevant adjustment variables suggests that the influences of race and poverty status on CVD risk may extend beyond education, literacy, substance use, depression, access to healthcare and select medical co-morbidities.

The current work also has important limitations. In that regard, the findings do not generalize to ethnic minority populations other than AAs. Furthermore, the results may be unique to the urban environment of Baltimore city. Importantly, only crude measures of health habits were obtained. More detailed information about the topography of smoking, alcohol, and illicit drug use should be informative in future work. We also did not have a physical activity measure. Next, although this work addressed the influence of poverty independent of education and literacy, we did not contrast respective influences of these SES indicators. Further, these SES factors may work in concert suggesting that future work should evaluate their combined influence. In addition, we did not have a full spectrum of SES indicators including occupational status or wealth. We did not test whether our adjustment variables operated as partial mediators of the relations of race and/or poverty status to CVD risk. Our CRP samples were assayed by two different institutions; although an internal reliability study was computed at the time of change, the associated data are not available. Our AA sample included a proportionally larger group of persons living in poverty than our White sample. Another limitation is the amount of missing data for each of the outcome variables. Lastly, we computed multiple comparisons without associated adjustment of *p* values; thus, the present findings (particularly interactions) should be interpreted with caution. In that regard, it is unlikely that the present sample is sufficiently large to support reasonable power for examining all of our interactions. Replication is necessary to establish firmer evidence for these effects. In addition, it will be important to examine whether noted associations differ as a function of age.

## Conclusion

Further understanding of the interactive and independent relations of race and SES to CVD risk factors, and their relevant underlying mechanisms, is critical to development of individual level and community based prevention and intervention efforts, in addition to relevant policy. The present findings suggest that poverty confers differential risk for higher BMI and WC, and lower HDL-C, among AAs and Whites. Elucidation of complex, multi-level mediators of these differential associations is needed as patterns of risk and resilience may vary within these subgroups of persons. The present findings further suggest that race-related patterns of CVD risk - better lipid profiles, but higher HbA1c and blood pressure in AAs compared to Whites – are not fully explained by a host of SES, behavioral, psychosocial, and biomedical adjustment variables. Continued identification of the micro-to-macro level mechanisms underlying these race differences will be critical to the elimination of health disparities. Future work would benefit from use of path analysis or structural equation modeling to explore whether mediational pathways differ among AA and White men and women who vary in SES.

## References

[1] Go AS (2013). Heart disease and stroke statistics - 2014 update. A report from the American Heart Association. Circulation.

[2] Williams DR, Mohammed SA, Leavell J, Collins C (2012). Race, socioeconomic status, and health: complexities, ongoing challenges, and research opportunities. Ann NY Acad Sci..

[3] Karlamangla AS, Merkin SS, Crimmins EM, Seeman TE (2010). Socioeconomic and ethnic disparities in risk for cardiovascular disease in the United States, 2001–2006. Ann Epidemiol.

[4] Adler NE, Rehkopf DH (2008). US disparities in health: descriptions, causes, and mechanisms. Annu Rev Publ Health.

[5] Gorelick PB (1998). Cerebrovascular disease in African Americans. Stroke.

[6] Kurian AK, Cardarelli KM (2007). Racial and ethnic differences in cardiovascular disease risk factors: a systematic review. Ethnic Dis.

[7] Williams DR (1997). Race and health: basic questions, emerging directions. Ann Epidemiol.

[8] Diez-Roux AV, Nieto FJ, Tyroler HA, Crum LD, Szklo M (1995). Social inequalities and atherosclerosis: the Atherosclerosis Risk in Communities Study. Am J Epidemiol.

[9] Fuller-Rowell TE, Curtis DS, Doan SN, Coe CL (2015). Racial disparities in the health benefits of educational attainment: a study of inflammatory trajectories among African American and White adults. Psychosom Med.

[10] Lewis TT, Everson-Rose SA, Sternfeld B, Karavolos K, Wesley D, Powell LH (2005). Race, education, and weight change in a biracial sample of women at midlife. Arch Intern Med.

[11] Knox SS, Jacobs DR, Chesney MA, Raczynski J, McCreath H (1996). Psychosocial factors and plasma lipids in black and white young adults: the Coronary Artery Risk Development in Young Adults Study data. Psychosom Med.

[12] Boykin S, Diez-Roux AV, Carnethon M, Shrager S, Ni H, Whitt-Glover M (2011). Racial/ethnic heterogeneity in the socioeconomic patterning of CVD risk factors: in the United States: the multi-ethnic study of atherosclerosis. J Health Care Poor U.

[13] Evans MK, Lepkowski JM, Powe NR, LaVeist T, Kuczmarski MF, Zonderman AB (2010). Healthy aging in neighborhoods of diversity across the life span (HANDLS): overcoming barriers to implementing a longitudinal, epidemiologic, urban study of health, race, and socioeconomic status. Ethnic Dis.

[14] Radloff LS (1977). The CES-D scale a self-report depression scale for research in the general population. Appl Psycho Meas.

[15] Wilkinson GS (1993). Wide Range Achievement Test, Revised (WRAT-3).

[16] Fradkin C, Wallander J (2014). Associations between socioeconomic status and obesity in diverse, young adolescents: variation across race/ethnicity and gender. Health Psychol.

[17] Freedman DS, Strogatz DS, Eaker E, Joesoef MR, DeStefano F (1990). Differences between black and white men in correlates of high density lipoprotein cholesterol. Am J Epidemiol.

[18] Farmer MM, Ferraro KF (2005). Are racial disparities in health conditional on socioeconomic status?. Soc Sci Med.

[19] Manly JJ (2006). Deconstructing race and ethnicity: implications for measurement of health outcomes. Med Care.

[20] Coleman-Jensen, Alisha, Mark Nord, Margaret Andrews, and Steven Carlson. Household Food Security in the United States in 2011. ERR-141, U.S. Department of Agriculture, Economic Research Service; 2012.

[21] Allen AJ, Kuczmarski MF, Evans MK, Zonderman AB, Waldstein SR. Race differences in diet quality of urban food-insecure blacks and white reveals resiliency in blacks. J Racial Ethn Health Disparities. 2015. Epub 10.1007/s40615-015-0189-5 PMC4911313 27294760

[22] Montez JK, Zajakova A (2013). Trends in mortality risk by education level and cause of death among US White women from 1986 to 2006. Am J Public Health.

[23] Borrell LN, Kiefe CI, Diez-Roux AV, Williams DR, Gordon-Larsen P (2013). Racial discrimination, racial/ethnic segregation and health behaviors in the CARDIA Study. Ethnic Health.

[24] Vines AI, Baird DD, McNeilly M, Hertz-Picciotto I, Light KC, Stevens J (2006). Social correlates of the chronic stress of perceived racism among Black women. Ethnic Dis.

[25] Groesz LM, McCoy S, Carl J, Saslow L, Stewart L, Stewart J, Adler N, Laraia B, Epel E. What is eating you? Stress and the drive to eat. Appetite. 2012;58:717–21. 10.1016/j.appet.2011.11.028 PMC3740553 22166677

[26] Lin SX, Carnethon M, Szklo M, Bertoni A (2011). Racial/ethnic differences in the association of triglycerides with other metabolic syndrome components: the Multi-Ethnic Study of Atherosclerosis. Metab Syndr Relat D.

[27] Third Report of the National Cholesterol Education Program (NCEP) Expert Panel on Detection, Evaluation, and Treatment of High Blood Cholesterol in Adults (Adult Treatment Panel III). Circulation. 2002;17:3143-3421. 12485966

[28] Adler NE, Stewart J (2010). Preface to the biology of disadvantage: socioeconomic status and health. Ann NY Acad Sci.

[29] VanderWeele TJ, Robinson WR (2014). On the causal interpretation of race in regressions adjusting for confounding and mediating variables. Epidemiology.

[30] Adler N, Bush NR, Panell MS (2012). Rigor, vigor, and the study of health disparities. P Natl A Sci.

[31] Braveman PA, Cubbin C, Egerter S, Chideya S, Marchi KS, Metzler M, Posner S. Socioeconomic status in health research: one size does not fit all. JAMA-J Am Med Assoc. 2005;294(22):2879–88. 10.1001/jama.294.22.2879 16352796

[32] Kelaher M, Paul S, Lambert H, Ahmad W, Smith GD (2008). The impact of different measures of socioeconomic position on the relationship between ethnicity and health. Ann Epidemiol.

[33] Allen AJ, McNeely JM, Waldstein SR, Evans MK, Zonderman AB (2014). Subjective socioeconomic status predicts Framingham cardiovascular disease risk in whites, not blacks. Ethn Dis.

[34] Braveman PA, Gottlieb L (2014). The social determinants of health: it’s time to consider the causes of the causes. Public Health Rep.

[35] Brondolo E (2015). Racial and ethnic disparities in health: examining the contexts that shape resilience and risk. Psychosom Med.

[36] Gallo LC, Matthews KA (2003). Understanding the association between socioeconomic status and physical health: do negative emotions play a role?. Psychol Bull.

